# Supplementary material for: The genome sequence of the mirid plant bug, Closterotomus trivialis (A.Costa, 1853) (Hemiptera: Miridae)
Source: Wellcome Open Res. 2025 Sep 15;10:514. [Version 1] doi: 10.12688/wellcomeopenres.24886.1 (PMC12521901; doi:10.12688/wellcomeopenres.24886.1)
Supplement: Peer review history

# Reviewer report: Reviewer response for version 1

**DOI:** 10.21956/wellcomeopenres.27413.r133366

**Published:** 2025-10-14

**Authors:** Zheng Li

**Affiliations:** 1 Miami University Department of Biology, Oxford, Ohio, USA

**The authors present a 1,163.70 megabase genome assembly of Closterotomus trivialis (mirid plant bug) using PacBio HiFi long reads and HIC sequencing data with total sequencing coverage of 32x. The genome is of high quality, with 94.03% percent of it localized on 16 chromosomal pseudomolecules. They also assembled the mitochondrial genome, which is approximately 19.42 kilobases in length. This genome represents a valuable resource for understanding the biology of an emerging agricultural pest species affecting citrus and olive production. It can also help in performing comparative genomic analysis to understand the evolutionary history of Miridae. The authors provided a solid background on sexual dimorphism, anatomical and life cycle distribution, and pest impact. The method and metadata are clear, and the results are well documented.**

**Here are some points that can be improved in the manuscript: **

- **In Figure 5, the assembly metrics for ihCloTriv1.1 are presented, including BUSCO statistics. However, the figure shows 3.31% of BUSCOs as missing, while Table 4 reports M: 2.3%. This discrepancy should be checked and clarified.**
- **While authors performed a BlobToolKit GC-coverage plot for ihCloTriv1.1, the authors did not include a cumulative sequence length plot, which is commonly presented in genome papers.**

Are sufficient details of methods and materials provided to allow replication by others?

Yes

Is the rationale for creating the dataset(s) clearly described?

Yes

Are the datasets clearly presented in a useable and accessible format?

Yes

Are the protocols appropriate and is the work technically sound?

Yes

Reviewer Expertise:

Genome evolution

I confirm that I have read this submission and believe that I have an appropriate level of expertise to confirm that it is of an acceptable scientific standard.

# Reviewer report: Reviewer response for version 1

**DOI:** 10.21956/wellcomeopenres.27413.r133362

**Published:** 2025-10-07

**Authors:** Roberto Biello

**Affiliations:** 1 John Innes Centre Department of Crop Genetics, Norwich, England, UK

The authors present a chromosome-level genome assembly for *Closterotomus trivialis*. This represents an important resource for studying the evolution and invasive potential of this pest species, which affects both citrus and olive trees. Sequencing was performed using PacBio HiFi and Hi-C technologies, and both primary and alternate assemblies were generated. The assembly meets high-quality standards, as demonstrated by high BUSCO scores and k-mer completeness. The methods are clearly described, and all associated data are publicly available.

 Minor comments:

 - Given that both HiFi and Hi-C data were available from the same individual, it would be interesting to understand why the authors chose to assemble the genome using Hifiasm with the --primary option, rather than performing a haplotype-resolved (diploid) assembly, which might have provided additional insights into genomic features such as structural variation.

 - The authors should clarify the sex of the specimen sequenced and indicate the expected sex chromosomes in this species, as this information is relevant for interpreting the assembly and chromosome composition.

Are sufficient details of methods and materials provided to allow replication by others?

Yes

Is the rationale for creating the dataset(s) clearly described?

Yes

Are the datasets clearly presented in a useable and accessible format?

Yes

Are the protocols appropriate and is the work technically sound?

Yes

Reviewer Expertise:

Genome assembly and annotation, Evolution, Genomics

I confirm that I have read this submission and believe that I have an appropriate level of expertise to confirm that it is of an acceptable scientific standard.
